# The Amphibian Major Histocompatibility Complex—A Review and Future Outlook

**DOI:** 10.1007/s00239-024-10223-7

**Published:** 2025-01-07

**Authors:** Joana Sabino-Pinto, Martine E. Maan

**Affiliations:** Groningen Institute for Evolutionary Life Sciences, University of Groningen, Groningen, Netherlands

**Keywords:** MHC, Amphibia, Immune system, Immunity, HLA

## Abstract

The major histocompatibility complex (MHC) is a cluster of functionally related genes encoding proteins which, among other functions, mediate immune system activation. While the MHC of many vertebrates has been extensively studied, less is known about the amphibian MHC. This represents an important knowledge gap because amphibians mark the evolutionary transition from an aquatic to a terrestrial lifestyle and often maintain a biphasic lifestyle. Hence, they tend to be exposed to both aquatic and terrestrial pathogen communities, providing opportunities to gain fundamental insights into how the immune system responds to different environmental challenges. Moreover, amphibians are globally threatened by invasive pathogens and the MHC may play a role in combating population decline. In this review, we summarize the current state of knowledge regarding the amphibian MHC and identify the major differences with other vertebrates. We also review how the number of MHC gene copies varies across amphibian groups and how MHC-based variation relates to amphibian ontogeny, behaviour, disease, and phylogeography. We conclude by identifying knowledge gaps and proposing priorities for future research.

**Supplementary Information:**

The online version contains supplementary material available at 10.1007/s00239-024-10223-7.

## Introduction

The immune system encompasses a plethora of defense mechanisms that protect the host from invading pathogens and other non-pathogenic diseases such as cancer (Kindt et al. 2007; Paul 2022). A well-functioning immune system detects a wide range of pathogens and rogue host cells, without attacking beneficial or harmless microflora that inhabit the host or the healthy tissues of the host itself (autoimmunity) (Kindt et al. 2007; Paul 2022). In the context of disease ecology, understanding host–pathogen dynamics (*e.g.*, infection outcomes) allows to disentangle infection (the pathogen entering the body) from disease (the organism incurring damage) and to potentially predict the need for intervention. With this review we shed light onto the amphibian major histocompatibility complex (MHC), because broadening our taxonomic scope can enhance our understanding of the evolution of the vertebrate immune system. Studying amphibians can be particularly rewarding in this context because they mark the evolutionary transition from an aquatic to a terrestrial lifestyle (entailing very different environments and pathogen threats), and they have maintained these two lifestyles in their present-day life-histories. In addition, understanding the amphibian immune system may contribute to the successful conservation of this highly threatened group.

Functionally, the immune system of vertebrates consists of two parts, the innate immune system and the acquired (or adaptive) immune system (Kindt et al. 2007; Paul 2022). The innate immune system provides the first line of defense against infection and comprises disease resistance mechanisms that are not targeted at a particular invader. It includes physical and chemical barriers that prevent pathogens from entering the organism (e.g. mucus) and that create micro-environments in which pathogens cannot survive (e.g. temperature), and cellular defenses (*e.g.*, macrophages) that ingest and destroy foreign materials. These are immediate responses that are active prior to, or right after, a pathogen entering the organism (Kindt et al. 2007; Paul 2022). On the other hand, the acquired immune system relies heavily on cellular responses (i.e., lymphocytes) and once stimulated can develop lifelong protection of the individual host by means of antibodies. Acquired immune responses are much slower (when compared to the innate responses) and can take several weeks to develop (in a first infection situation—where the organism generates antibodies to deal with a particular pathogen); they are specifically relevant (and much faster) in re-infection scenarios (Kindt et al. 2007; Paul 2022). Whereas the innate immune system exists in all organisms, the acquired immune system is hypothesized to have evolved around 500 million years ago, with the divergence of the jawed vertebrates from the jawless fish (Flajnik 2018), and has become a key characteristic of vertebrate immune protection. Together, the two systems form complementary lines of defense: the innate immune system presents a permanent and immediate, while non-specific, response to invaders that delays the invasion, while the acquired immune system presents a slow and highly specific response to target and kill those invaders.

The immune system of the jawed vertebrates (both innate and acquired) relies largely on the MHC (Murphy et al. 2022). The MHC region is a cluster of functionally related genes that, among other functions, are involved in self vs. non-self recognition (Murphy et al. 2022). In other words, MHC gene products play a role in distinguishing between host and 'foreign' entities, which makes the MHC a key player in jawed vertebrates’ disease dynamics. These proteins present antigens of invading pathogens to the host immune system allowing for the development of an appropriate response (Kindt et al. 2007; Paul 2022). The MHC is a common focus area in studies of vertebrate population declines due to genetic erosion (i.e., loss of genetic diversity), especially when diseases are involved (Bernatchez and Landry 2003), as lower MHC diversity may threaten population persistence (but see Babik et al. 2009; Höglund et al. 2015). The role of MHC genes in individual fitness and evolutionary adaptation is well established in many vertebrate species, particularly in fish, birds, and mammals (Bernatchez and Landry 2003). Much less is known about other groups, such as amphibians. This is particularly striking as amphibians are not only exposed to a wide variety of pathogens but are also currently threatened with extinction because of some of those pathogens. In this review, we aim to summarize the current state of knowledge regarding the amphibian MHC, particularly the antigen-presenting genes. We begin by providing a general description of the structure and function of the MHC. Subsequently, we summarize observed patterns of variation in relation to amphibian geography, phylogeny, life history, and behavior. By identifying knowledge gaps and proposing directions for future research, we hope to contribute to a better understanding of the evolution of vertebrate immunity as well as serve amphibian conservation.

### The Major Histocompatibility Complex

**Fig. 1:**
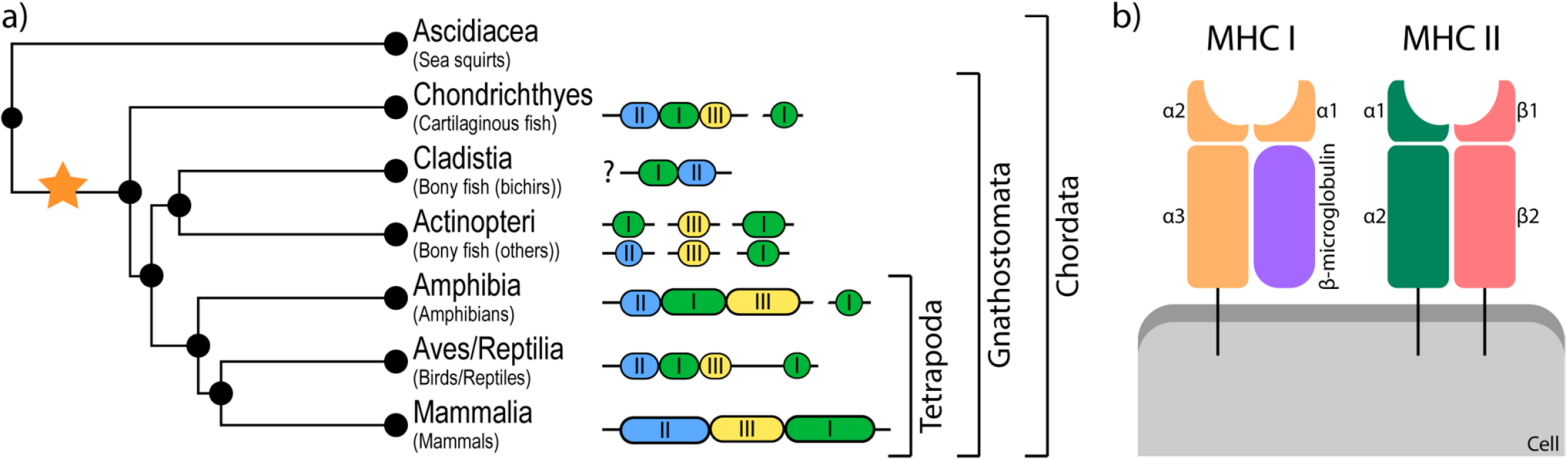
MHC organization and structure. **a** Simplified representation of the MHC genomic organization across the Gnathostomata (jawed vertebrates). The star marks the origin of the MHC. The question mark indicates that for the Cladistia, knowledge of MHC organization is limited to linkage between MHC class I and II genes (Dijkstra et al. 2013). Data extracted from (Flajnik and Kasahara 2001; Belov et al. 2006). **b** Schematic representation (not to scale) of the MHC class I and class II glycoproteins and their positioning on a cell, with the different protein domains. Phylogenetic tree was created with timetree.org, figure was created with Adobe Illustrator

The MHC region comprises three subregions: MHC class I, MHC class II and MHC class III, and encodes about 200 genes in mammals and amphibians (The MHC sequencing consortium 1999; Ohta et al. 2006) and even more in fish and birds (Sambrook et al. 2005; Star et al. 2011). Genes from MHC classes I and II subregions encode proteins related to antigen presentation (*e.g.*, proteins involved in peptide binding and antigen processing) with conserved structures (Fig. 1b), while genes from the MHC class III subregion encode proteins with other immune functions, (*e.g.*, complement factors, immune signalling) and a wide variety of protein structures (Kindt et al. 2007). Across jawed vertebrates, including amphibians, the positioning of these subregions in the genome typically has MHC class II genes tightly linked to MHC class I genes, followed by MHC class III genes (Fig. 1a) (Flajnik and Kasahara 2001; He et al. 2023).

Some MHC genes (both class I and II) encode transmembrane glycoproteins that mediate self vs. nonself recognition (Murphy et al. 2022). These genes emerged with the jawed vertebrates (Fig. 1a) and have a conserved function (Kasahara et al. 1995; Bernatchez and Landry 2003). The proteins they generate have a transmembrane domain that anchors the protein on the cell membrane, and an exposed part that binds to antigens and presents them to T lymphocytes (Fig. 1b) (Penn 2005). Due to the direct effects of these proteins in immunity, variation in the genes that encode these proteins is essential, placing these genes among the most polymorphic loci known in vertebrates (Sommer 2005). MHC class I and II proteins are generally divided into two subcategories, classical and non-classical (not to be confused with the α/β protein domains shown in Fig. 1b), based on their structure and function (reviewed in Paul 2022):

- Classical MHC class I (or MHC Ia) proteins are highly diverse molecules that are expressed on the cell surface of most nucleated host cells. They mostly present short peptides to T lymphocytes that result from the intracellular digestion of pathogens (usually viruses or intracellular bacteria) (but see Purnima et al. 2017). When T lymphocytes interact with these MHC proteins, and recognize the presented peptide as foreign, they initiate a cytotoxic response that destroys the infected cell (reviewed in Paul 2022).
- Non-classical MHC class I (or MHC Ib and XNC) proteins are characterized by low abundance and low diversity (in comparison to MHC Ia) (Hughes et al. 1999) and can be found on the cell surface of most cells (Adams and Luoma 2013). Their function is less well understood, but many of them present specific peptides and non-peptide antigens to both T lymphocytes and natural killer cells (Stroynowski and Lindahl 1994; Adams and Luoma 2013).
- Classical MHC class II proteins are present on the membranes of professional antigen presenting cells (*i.e.*, macrophages, B lymphocytes, and dendritic cells) and mostly present slightly larger peptides (than MHC Ia) to T lymphocytes that result from the degradation of phagocyted particles (*e.g.*, extracellular pathogens, such as bacteria and fungi) (but see Purnima et al. 2017). When T lymphocytes interact with these MHC proteins (and recognize the presented protein as foreign), they recruit cytokines and activate other immune cells, initiating acquired immune cascade responses (reviewed in Paul 2022).
- Non-classical MHC class II (DM and DO) proteins are present in internal membranes of professional antigen presenting cells (*i.e.*, macrophages, B lymphocytes, and dendritic cells) and have a role in the loading of peptides onto classic MHC class II molecules (Alfonso and Karlsson 2000).

Together, these MHC proteins protect vertebrates against a diverse array of pathogens. Each MHC class I and II protein binds to different peptide sequences, with different proteins interacting with different pathogens (Kaufman 2018). The binding ability is determined by the amino-acid composition of the MHC molecule’s peptide binding region (PBR), in terms of both sequence and motif (folding structure) (Piertney and Oliver 2006). This means that sequence variation at the PBR is crucial for the host’s ability to recognize and fight multiple pathogens (Sommer 2005; Kosch et al. 2019). Yet, optimal MHC allelic diversity is lower than maximal MHC allelic diversity at these sites (Woelfing et al. 2009): high allelic variation in an individual is not necessarily beneficial. It is hypothesized that this phenomenon is a consequence of the maturation process of T lymphocytes. During this process, T lymphocytes are checked by the organism to ensure that they do not interact with MHC proteins presenting self-peptides to avoid auto-immunity. As a result, higher numbers of MHC alleles increase the probability for T-lymphocytes to be rejected (Nowak et al. 1992; Woelfing et al. 2009). MHC class I and II allelic diversity is used as an indicator of the immunological health of vertebrate populations (Parham 2005), although this assertion is debated as not all populations with low MHC allelic diversity have reduced survival (Radwan et al. 2010).

MHC class I and II polymorphism is achieved through allelic diversity (Bjorkman et al. 1987) and/or copy number variation (Schaschl et al. 2009; Siddle et al. 2010). Mechanisms that generate new alleles and gene copies include: mutation, duplication, recombination, and introgression (Edwards and Hedrick 1998; Richman et al. 2003; Dudek et al. 2019). The same allele can also generate different peptides when specific exons are selectively removed or inserted in the messenger RNA, resulting in shorter or longer molecules (*i.e.*, alternative splicing; reviewed in Belicha-Villanueva et al. 2010; Paul 2022). This process can also create truncated non-functional proteins that either rapidly degrade or compete with the properly spliced variants.

The mechanisms mentioned above operate under both selective and non-selective pressures. For instance, natural selection may favour individuals with specific MHC genotypes by increasing their likelihood of survival and reproduction (Milinski 2006). This selective pressure can also extend to sexual selection. For example, individuals may prefer mating partners carrying different MHC alleles than themselves, thereby promoting MHC diversity at the population level, as predicted by theoretical models (*e.g.*, Ejsmond et al. 2014). Non-selective pressures such as bottlenecks and founder effects can reduce the number of alleles in a population (Ploshnitsa et al. 2012). This is commonly observed in small and fragmented populations, such as northern amphibian populations in the northern hemisphere (Höglund et al. 2022). Selective and non-selective pressures act in tandem, collectively influencing the allele frequencies within populations (Höglund et al. 2015).

### Amphibians: A Brief Introduction

Amphibians are ectotherms that mark the evolutionary transition from water to land. They are generally characterized by having naked skin, aquatic larvae, and terrestrial adults (biphasic lifestyle), although some species develop directly from egg to adult (Duellman and Trueb 1994). They present a wide variety of life histories, even among closely related species, but many species are nocturnal omnivores that live in close association with water (Duellman and Trueb 1994; Oliveira et al. 2017). Phylogenetically, amphibians are divided into three orders: (1) Anura, the tailless amphibians, including frogs and toads; (2) Caudata, the tailed amphibians, including salamanders and newts; and (3) Gymnophiona, the limbless amphibians, including caecilians (Duellman and Trueb 1994). Currently there are over 8800 amphibian species described, with 88% of those species belonging to the order Anura, 9% belonging to the order Caudata, and only 3% belonging to the order Gymnophiona (Frost 2023).

Amphibians are extremely important in many ecosystems, both as voracious consumers (of algae and invertebrates in the tadpole stage, and of small (in)vertebrates as adults) and as prey of, for example, dragonflies, spiders, birds, and mammals (Duellman and Trueb 1994; Touchon and Vonesh 2016). They are considered sentinel species due to their sensitive physiology which makes them vulnerable to environmental stressors (e.g., pollution and temperature fluctuations) (Burkhart et al. 1999; Burlibaşa and Gavrilacaron; 2011; Thammachoti 2012). In addition to their intrinsic value as unique and charismatic inhabitants of planet Earth, they also confer various economical and societal benefits to humans. For instance, as food source (Kyselý 2008; Ribeiro et al. 2019), traditional pregnancy tests (Elkan 1938), compounds for drug development (Conlon and Sonnevend 2011), and natural pesticides (Mohneke and Rödel 2009).

Since the 1980s amphibians have been experiencing drastic worldwide declines (Luedtke et al. 2023). According to the most recent IUCN report, more than a third of the 5915 amphibian species assessed—out of the 6041 species described at the time—are threatened with extinction and at least 34 of those have gone extinct in the last ~ 500 years (Stuart et al. 2008). This makes amphibians the most severely threatened animal group (IUCN 2022) with an extinction rate that substantially surpasses the estimated historical background extinction rate (McCallum 2007; Roelants et al. 2007). Several causes have been identified for these declines, including habitat destruction and emerging infectious diseases (Luedtke et al. 2023). Given the latter, a thorough understanding of the amphibian immune system is essential for mitigating pathogen-mediated threats (Pabijan et al. 2020). From a more fundamental perspective, amphibians are a rewarding model system for studying immunity in general (Liu et al. 2016) because of their interesting phylogenetic position (transition between aquatic and terrestrial vertebrates), biphasic lifestyle (exposing them to both terrestrial and aquatic pathogens), and the independent repeated evolution of water-independent histories.

## The Amphibian MHC: Structure

**Fig. 2:**
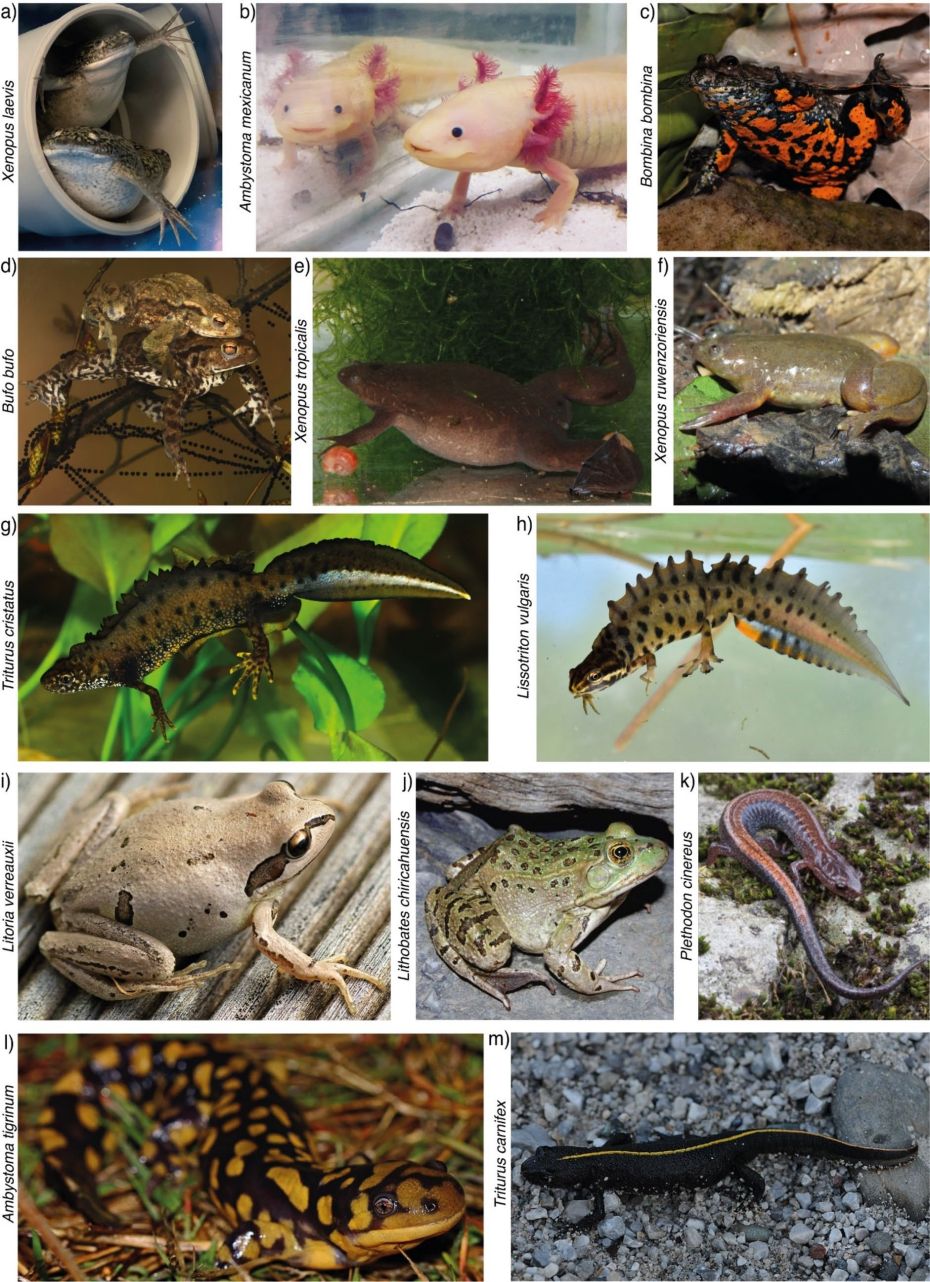
Examples of amphibians in which the MHC has been studied. **a** *Xenopus laevis* (South African clawed frog); **b** *Ambystoma mexicanum* (Axolotl); **c** *Bombina bombina* (European fire-bellied toad); **d** *Bufo bufo* (Common toad); **e** *Xenopus tropicalis* (Western clawed frog); **f** *Xenopus ruwenzoriensis* (Uganda clawed frog); **g** *Triturus cristatus* (Northern crested newt); **h** *Lissotriton vulgaris* (Smooth newt); **i** *Litoria verreauxii* (Whistling tree frog); **j** *Lithobates chiricahuensis* (Chiricahua leopard frog); **k** *Plethodon cinereus* (Red-backed salamander); **l** *Ambystoma tigrinum* (Tiger salamander); **m** *Triturus carnifex* (Italian crested newt). Figure was created with Adobe Illustrator. [Photo credits: **a** Julia Kamenz; **b** Katharina Ruthsatz; **c** Miguel Vences; **d** Miguel Vences; **e** Václav Gvoždík; **f** Gauvain Saucy; **g** Miguel Vences; **h** Miguel Vences; **i** Ian Sutton; **j** Jim Rorabaugh; **k** Bill Peterman; **l** Peter Paplanus; **m** Böhringer Friedric.]

The amphibian MHC was first identified in 1975 (Roux and Volpe 1975), but until recently, our knowledge was largely based on only two model species, *Xenopus laevis* (South African clawed frog; Fig. 2a) (Anura: Du Pasquier et al. 1989; Flajnik 2018) and *Ambystoma mexicanum* (Axolotl; Fig. 2b) (*Caudata*: Sammut et al. 1997; Völk et al. 1998). The number of amphibian taxa in which the MHC has been studied has now grown to 92 species belonging to 32 genera (Online Resource 1). This represents ~ 1% of all known amphibian species (92 out of 8827) (Frost 2023) and is strongly biased towards anurans (Williams et al. 2020; Online Resource 1).

The amphibian immune system is structurally and functionally similar to that of other vertebrates (reviewed in Du Pasquier et al. 1989; Robert and Ohta 2009), but there are some key differences. (1) Many amphibians (when compared to mammals) have one additional codon in the classical MHC class II PBR. This codon has been found to be subject to positive selection in multiple species of Ranidae, Bufonidae, and Leiopelmatidae (Bataille et al. 2015; Lillie et al. 2015; Mulder et al. 2017), and while its function is unknown, the signature of selection suggests that it might be important in pathogen recognition (Mulder et al. 2017). (2) In the frog *X. laevis*, MHC class II proteins are present on cells of the myeloid lineage (*e.g.*, monocytes and macrophages) as well as on T lymphocytes and epithelial cells (Du Pasquier and Flajnik 1990; Rollins-Smith and Blair 1990; Salter-Cid and Flajnik 1995), instead of being restricted to specialized antigen presenting cells (*e.g.*, dendritic cells and B lymphocytes) as in mammals (Murphy et al. 2022). (3) MHC introns are longer in amphibians than in mammals and birds (He et al. 2023). (4) Unlike in mammals, non-classical MHC class I codons appear to be highly conserved in the one amphibian genus studied (*Xenopus*, Flajnik et al. 1993; Goyos et al. 2011; Edholm et al. 2014), with a high degree of similarity between species that diverged over 50 Mya (Edholm et al. 2014). (5) Some genes are found in different MHC class regions than in mammals (Kato et al. 1994; Salter-Cid et al. 1994, 1996; Namikawa et al. 1995; Ohta et al. 1999).

Between amphibian lineages there are also differences in MHC gene diversity and organization (He et al. 2023). For instance, the non-classical MHC class I genes lie outside the MHC cluster in *X. laevis* (the only studied Anuran representative; Courtet et al. 2001) but are physically linked to the classical MHC class I and class II genes in *A. mexicanum* (the only studied Caudata representative; Sammut et al. 1999). It must be noted, however, that some aspects of the MHC genomic organization in *X. laevis* and *A. mexicanum* are subject to debate (*i.e.*, studies disagree as to whether MHC class I and II regions are connected or separated by the MHC class III region, Sammut et al. 1999; Schloissnig et al. 2021; He et al. 2023).

## MHC Copy Number Variation in Amphibians

Amphibians tend to possess a larger number of functional **classical MHC class I** genes than mammals (Amphibians: 1–21 Sammut et al. 1999; Trujillo et al. 2021, Online Resource 1); Mammals: 1–4 (Pan et al. 2008; Zhu et al. 2013)), but fewer than birds and fish (Birds: 1–33 (Minias et al. 2019); Fish: < 100 (Star et al. 2011)). Functional interpretation of MHC gene copy numbers is not straightforward. For instance, the relatively high number of MHC class I gene copies observed in some fish species may serve as a compensatory mechanism for their lack of MHC class II genes (Star et al. 2011). Interestingly, classical MHC class I gene diversity in salamanders is positively correlated with that of some antigen processing genes, suggesting co-evolution between these two groups of genes (Palomar et al. 2021).

**Fig. 3:**
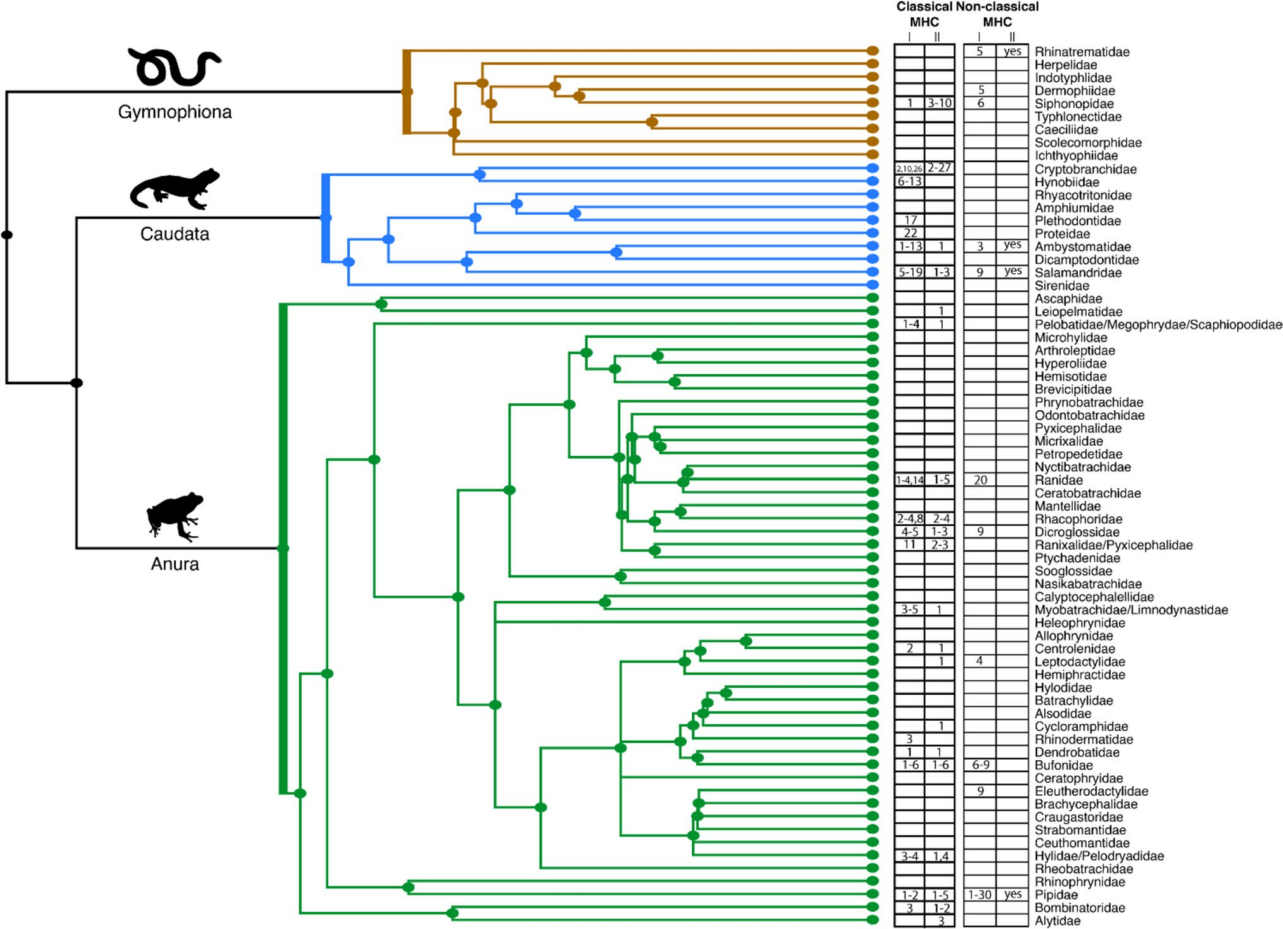
Variation of MHC gene copy numbers across amphibians. Amphibian phylogenetic tree at the family level with the numbers of classical and non-classical MHC class I and class II gene copies reported for the studied families. For sources see Table 1 and Online Resource 1. Phylogenetic tree was created with timetree.org, figure was created with Adobe Illustrator

Amphibians appear to have fewer classical MHC class II gene copies than any other vertebrate group (Mikko et al. 1999; Wilson 2017; Savage et al. 2020). At present, the evolutionary mechanisms underlying this disparity are unknown, but it could be related to the reliance of amphibians on non-classical MHC class I genes. The number of classical MHC class II gene copies also tends to be lower than the number of MHC class I gene copies (Fig. 3, Table 1), suggesting differential selection pressures between MHC classes.

**Table 1:** Estimated numbers of MHC gene copies in amphibians. No information is available for non-classical MHC class I. For more details see Online Resource 1

		Classical		Non-classical	References
		MHC I	MHC II	MHC II	
*Anura*	*Alytidae*	–	3	–	(Hauswaldt et al. 2007)
	*Bombinatoridae*	3	2 and 3	–	(Hauswaldt et al. 2007; Schröder et al. 2012; Zhao et al. 2014; Bataille et al. 2015; Talarico et al. 2018)
	*Bufonidae*	1 and 4	1 to 6	1	(May and Beebee 2009; Kiemnec-Tyburczy et al. 2010; May et al. 2011; Zeisset and Beebee 2013b, 2014; Lillie et al. 2016, 2014; Bataille et al. 2015; Höglund et al. 2015, 2022; Didinger et al. 2017; Carlson et al. 2022; Cortazar-Chinarro et al. 2022; Dearborn et al. 2022; Hase 2022)
	*Centrolenidae*	2	1	–	(Kiemnec-Tyburczy et al. 2012; Kiemnec-Tyburczy and Zamudio 2012)
	*Cycloramphidae*	–	1	–	(Belasen et al. 2019)
	*Dicroglossidae*	–	2 and 3	–	(Yu et al. 2014; Hu et al. 2017)
	*Hylidae*	3 and 4	1	–	(Kiemnec-Tyburczy et al. 2012, 2018; Bataille et al. 2015; Didinger et al. 2017; Fu et al. 2023b)
	*Leiopelmatidae*	–	1	–	(Lillie et al. 2015)
	*Leptodactylidae*	–	1	–	(Kosch et al. 2016)
	*Megophryidae*	4	–	–	(Zhang et al. 2015)
	*Myobatrachidae*	4 and 5	–	–	(Kosch et al. 2017, 2019)
	*Pipidae*	1 and 2	1 to 3	2	(Du Pasquier et al. 1977; Obara et al. 1983; Flajnik et al. 1984, 1991, 1999; Kaufman et al. 1985a; Sato et al. 1993; Shum et al. 1993; Kobari et al. 1995; Sammut et al. 2002; Liu et al. 2002; Ohta et al. 2006; Mable et al. 2015)
	*Ranidae*	1 to 3	1 to 5	–	(Hauswaldt et al. 2007; Teacher et al. 2009; Zeisset and Beebee 2009; Kiemnec-Tyburczy et al. 2010, 2012; Savage and Zamudio 2011, 2016; Shu et al. 2013; Gong et al. 2013; Marosi et al. 2014; Lau et al. 2016, 2017; Wang et al. 2017; Mulder et al. 2017; Liu et al. 2017; Cortázar-Chinarro et al. 2017, 2018; Chen et al. 2018a; Savage et al. 2018, 2019, 2020; Hernández-Gómez et al. 2019; Trujillo et al. 2021)
	*Rhacophoridae*	2 and 3	2 to 4	–	(Zhao et al. 2013; Huang et al. 2016; Luo et al. 2016; Li et al. 2016; Chen et al. 2019)
*Caudata*	*Ambystomatidae*	1 to 19	1	–	(Sammut et al. 1997, 1999; Bos and DeWoody 2005; Richman et al. 2007; Bulut et al. 2008; Bos et al. 2009; Tracy et al. 2015; Williams et al. 2020; Minias et al. 2022)
	*Cryptobranchidae*	2 and 10	2 and 3	–	(Zhu et al. 2014; Hernández-Gómez et al. 2018; Dearborn et al. 2022; Minias et al. 2022)
	*Hynobiidae*	6 to 13	–	–	(Minias et al. 2022)
	*Plethodontidae*	17	–	–	(Minias et al. 2022)
	*Proteidae*	22	–	–	(Minias et al. 2022)
	*Salamandridae*	1 to 19	1 to 3	–	(Babik et al. 2008, 2009; Nadachowska-Brzyska et al. 2012; Wielstra et al. 2015; Dudek et al. 2019; Talarico et al. 2019; Minias et al. 2022; Gaczorek et al. 2023)

Amphibians tend to have relatively few non-classical MHC class I gene copies, apart from *Xenopus* in which about 30 gene copies can be found (Fig. 3). The high number of these genes in *Xenopus* was thought to be a compensatory mechanism for the low number of classical MHC class I genes that they possess (Ruiz and Robert 2023), however, other amphibians with few classical MHC class I genes have been shown to also possess few non-classical MHC class I genes (Fig. 3).

The number of classical MHC gene copies varies among amphibian families, with no discernible pattern across amphibian groups or between MHC classes (Fig. 3, Table 1, Online Resource 1). There is, however, evidence for an association between the number of classical MHC class I genes and life history in Caudata, where species with shorter lifespans tend to possess more classical MHC class I gene copies (Minias et al. 2022). This pattern was hypothesized to have evolved as a mechanism that reduces the vulnerability of fast-lived species to stochastic events, such as temporal/spatial changes in pathogen communities (Minias et al. 2022). The absence of an overall phylogenetic pattern in of the number of MHC gene copies might be attributed to the limited number of studied species, highlighting the need for expanding sampling efforts across the amphibian tree. For instance, Gymnophiona remains vastly unstudied (Fig. 3, Table 1, Online Resource 1). This gap is likely to be addressed in the near future, as new genomic data is currently generated, and organized efforts such as the Amphibian Genomics Consortium (Kosch et al. 2024) are underway to sequence the genomes of more amphibian species.

The number of classical MHC class I and II genes vary even within populations of the same species (Online Resource 1). For example, in *Bombina* and *Bufo* (Fig. 2c-d), some populations possess two classical MHC class II gene copies, while others have only one (Hauswaldt et al. 2007; May et al. 2011; Talarico et al. 2018). This variation may result from processes like duplication, deletion, or other types of mutations, although some of it could be attributed to methodological limitations. For instance, strict sequence grouping and allele selection thresholds might misclassify rare alleles as sequencing errors. Additionally, gene copy numbers can be underestimated due to allele sharing across loci (the number of gene copies is estimated by dividing the maximum number of alleles found in an individual by two, accounting for diploidy). Therefore, reported numbers of MHC class I and class II gene copies should be considered as minimum rather than actual counts. Overestimation of the number of gene copies can also occur, through the co-amplification of non-classical MHC genes or pseudogenes (Flajnik et al. 1993). To accurately determine copy numbers, it is essential to employ techniques beyond amplicon sequencing, such as whole-genome sequencing, long-read sequencing (PACBIO or Nanopore), or qPCRs (Solomon et al. 2008).

For an individual, maximal MHC diversity confers an increased risk of auto-immune reactions (Lawlor et al. 1990; Takahata 1995). In the *Xenopus* genus, which includes species with various chromosome counts, *Xenopus tropicalis* (Western clawed frog; Fig. 2e) is the true diploid with 20 chromosomes (Kobel and Du Pasquier 1986), one MHC class I gene copy and up to two MHC class II gene copies (Sato et al. 1993; Shum et al. 1993; Ohta et al. 2006). Polyploid species originating from whole-genome duplications do not necessarily have more MHC gene copies. This is the case in *X. ruwenzoriensis* (Uganda clawed frog; Fig. 2f), which has 108 chromosomes instead of the ancestral 20, that only has two classical MHC class I gene copies and three classical MHC class II gene copies (Sato et al. 1993; Sammut et al. 2002)—while maintaining a high number of gene copies on the remainder of the genome (Kobel and Du Pasquier 1986). This shows that polyploidy is not necessarily associated with an increased number of MHC gene copies (Du Pasquier and Blomberg 1982; Du Pasquier et al. 2009) and suggests a selective advantage for reducing the number of functional MHC genes.

## Geographic Distribution of Amphibian MHC Diversity

In jawed vertebrates, there is a general pattern of greater classical MHC allelic diversity in populations closer to the equator and reduced diversity in populations closer to the poles, traditionally called “southern richness and northern purity” (Hewitt 2000). This pattern is hypothesized to originate from the loss of genetic variation during range expansion out of glacial refugia (Hewitt 2004) and/or more intense pathogen-mediated selection in warmer regions (*i.e.*, parasite diversity increases towards the equator; Guernier et al. 2004). This pattern is also observed in amphibians (Table 2) and coincides with stronger signatures of balancing selection in populations closer to the equator and stronger signatures of drift in populations closer to the poles (Cortázar-Chinarro et al. 2018). In *Triturus cristatus* (Northern crested newt; Fig. 2g) for instance, that ranges from the Balkans to Scandinavia, the southern populations have 24 classical MHC class II alleles, and the northern ones only have two (Babik et al. 2009). An exception is *Lissotriton vulgaris* (Smooth newt; Fig. 2h), in which classical MHC allelic diversity is lower closer to the equator. In this species the higher diversity observed in populations closer to the poles was associated with introgression between species during post-glacial recolonizations (Nadachowska-Brzyska et al. 2012). The implications of this variation in allelic diversity across populations for immune functioning and local adaptation are unknown. With additional species studied, more exceptions may be observed, and such consequences are better understood.

**Table 2:** Studies documenting associations between amphibian geographic distribution and MHC protein presence and diversity. No information is available for non-classical MHC. For more details see Online Resource 1

	Classical		References
	MHC I	MHC II	
Higher latitude	More gene copies	Low diversity (purity)	(Babik et al. 2009; Wielstra et al. 2015; Lillie et al. 2015; Cortázar-Chinarro et al. 2017; Cortazar-Chinarro et al. 2022; Höglund et al. 2022; Minias et al. 2022)
Lower latitude	–	Diversity (purity in one study)	(Babik et al. 2009; Nadachowska-Brzyska et al. 2012; Zeisset and Beebee 2014; Wielstra et al. 2015; Talarico et al. 2018; Cortázar-Chinarro et al. 2018)
Altitude	Diversity	–	(Kiemnec-Tyburczy et al. 2018)
Isolation	More diverse alleles/fewer alleles/No isolation by distance/Diversity similar to high latitude populations	Fewer alleles / Same heterozygosity / (no) Isolation by distance / Genetic erosion / Shared alleles between populations	(Zeisset and Beebee 2010; Savage and Zamudio 2011; Höglund et al. 2015; Li et al. 2016; Wang et al. 2017; Kiemnec-Tyburczy et al. 2018; Belasen et al. 2019, 2022; Hernández-Gómez et al. 2019; Talarico et al. 2019; De Cahsan et al. 2021)
Translocation	–	Incorporation of local alleles in translocated populations	(Schröder et al. 2012; Zeisset and Beebee 2013a)
Invasion	Reduced diversity in expansion front and in invasion range, compared to native range	Same diversity at expansion front and in invasive populations	(Lillie et al. 2017; LaFond et al. 2022)
Time	Old lineages	Decrease in allelic richness over the years	(Flajnik et al. 1999; Trujillo et al. 2021)
Other	Similar genotypes between populations. Allelic tree follows species tree. Introgression	Similar genotypes between populations. No relation to range size. Introgression	(Tracy et al. 2015; Fijarczyk et al. 2018; Dudek et al. 2019; Belasen et al. 2021; De Cahsan et al. 2021; Williams et al. 2021; Gaczorek et al. 2023)

Classical MHC class I and II allelic diversity in amphibians is also affected by population range size (Table 2). For instance, small populations from islands tend to have reduced diversity when compared to their larger mainland counterparts, likely due to drift and isolation (Höglund et al. 2015; Belasen et al. 2019; Table 2) or perhaps due to lower pathogen pressures. Lower diversity is sometimes observed in populations from range expansion fronts (Lillie et al. 2017), but not always (LaFond et al. 2022). Overall, there is a tendency for populations with small distribution ranges to harbour fewer alleles while at the same time exhibiting higher intra-individual diversity (*i.e.*, higher levels of heterozygosity; Table 2; Wang et al. 2017; Kiemnec-Tyburczy et al. 2018). This relatively high level of intra-individual diversity may contribute to the maintenance of genetic diversity and population persistence in small populations.

Identical alleles are sometimes found in multiple species, through either convergence, introgression, or incomplete lineage sorting (trans-species polymorphism (TSP)) (Klein et al. 1998). In amphibians, sharing of classical MHC class I and II alleles across species has been associated with introgression (Zieliński et al. 2013; Dudek et al. 2019) and TSP (Bos & Waldman 2006b; Online Resource 1), but not as much with convergence (Fu et al. 2023a), likely due to a lack of data. This widespread allele sharing in amphibians is noteworthy. It is observed in 88% of MHC class I studies and in 65% of MHC class II studies (Online Resource 1; Kiemnec-Tyburczy et al. 2010; Tracy et al. 2015). Some of the alleles shared between species have been shown to play a critical role in the resistance to specific pathogens. For example, the allele Rapi_33 in *Rana pipiens* (Northern leopard frog) and *Rana catesbeiana* (American bullfrog) has been associated with disease tolerance to chytridiomycosis (Trujillo et al. 2021; LaFond et al. 2022). In the context of species conservation, classical MHC class I and II introgression is used as a tool: individuals can be actively translocated in order to introduce locally advantageous classical MHC alleles to boost adaptation to local pathogens (Schröder et al. 2012; Zeisset and Beebee 2013a). Such interventions do entail the hypothetical risk that introduced MHC alleles (or other genetic variation) can be maladaptive in the new environment and lead to increased susceptibility to other local pathogens, outbreeding depression, or immunopathology (inappropriate immune response to an infection) (Kubinak et al. 2012).

Amphibian populations that survive demographic crashes (*e.g.*, bottlenecks, fragmentation) do not seem to suffer drastic losses in classical MHC class I and II allelic diversity (Tracy et al. 2015; Kosch et al. 2017; but see Belasen et al. 2022; Becker et al. 2023), which hints at the operation of strong positive/balancing selection. However, while a larger classical MHC allelic diversity might be favourable in the long term (Radwan et al. 2010), there are MHC-depleted populations that do not seem to suffer any consequences (Babik et al. 2009; Höglund et al. 2015). For instance, *T. cristatus* from the postglacial expansion range in Europe have extremely low diversity in classical MHC class II alleles but have nevertheless survived for over 10,000 years (Babik et al. 2009). Possibly, the alleles present are adequate, or perhaps the classical MHC class II gene is not relevant for resistance against local pathogens. The fact that populations can persist with low allelic diversity makes it difficult to predict when and where allelic depletion will translate into population declines.

## MHC Expression During Amphibian Ontogeny

Many amphibians have two distinct life stages (*i.e.*, tadpole/larvae and adult) that are connected by the process of metamorphosis (Duellman and Trueb 1994). The amphibian immune system undergoes major changes during ontogeny (reviewed in Rollins-Smith 1998; Fort et al. 2007; Rollins-Smith and Woodhams 2012), with a steeper development in Anura and more gradual development in *Caudata* (Rollins-Smith 1998). In Anura, classical MHC class I proteins are virtually absent in pre-metamorphic tadpoles (Table 3; Salter-Cid et al. 1998; Pollet 2010; Lau et al. 2020), while in the only *Caudata* representative studied (*A. mexicanum*) classical MHC class I genes are expressed from hatching (Sammut et al. 1997; Tournefier et al. 1998; Table 3). In *Xenopus*, the lack of classical MHC class I gene expression in tadpoles seems to be compensated for by the expression of non-classical MHC class I genes (Goyos et al. 2009; Edholm et al. 2015, 2017). This is further supported by the observations that non-classical MHC class I genes contribute to immunity during the early life stages (Edholm et al. 2013, 2018) and are essential for successful development of young tadpoles (Banach et al. 2017). However, this abundance of non-classical MHC class I genes is not observed in other amphibians (Fig. 3; He et al. 2023). During metamorphosis, larval cells are replaced by adult cells with different MHC expression profiles (Izutsu et al. 2000). The appearance of classical MHC class I expression during metamorphosis is linked to the apoptosis of larval cells, as cells without it (*i.e.*, larval cells) are destroyed by the mature immune system (Table 3; Du Pasquier et al. 1979; Ruben et al. 1994; Izutsu et al. 1996). In Anura, expression levels of both classical MHC class I and II genes increase around the metamorphic climax (Lau et al. 2020), with different onsets of the increase in different tissues (Flajnik et al. 1990; Rollins-Smith et al. 1997; Salter-Cid et al. 1998; Table 3). This is documented in *X. laevis*, in which classical MHC class II expression is detected first in the thymus epithelium halfway through the tadpole stage but is only detected in B lymphocytes and mature T lymphocytes emerging from the thymus during metamorphosis (Du Pasquier and Flajnik 1990). This increase in classical MHC class II expression around metamorphosis coincides with the beginning of the transformation of the tadpole immune system (for a review see Rollins-Smith 1998). For generalizations, the observation that MHC gene expression changes over ontogeny (Didinger et al. 2017; Lau et al. 2020) and across tissues (Stewart et al. 2005; Savage et al. 2014; Table 3). The environment in which an individual develops also influences MHC expression. For instance, exposure to chemical pollutants reduces classical MHC class II surface expression (*X. laevis*; Robert et al. 2019).

**Table 3:** Studies documenting MHC protein expression across amphibian ontogeny. For more details see Online Resource 1

	Classical		Non-classical		References
	MHC I	MHC II	MHC I	MHC II	
Anura					
Tadpole	Low expression in early tadpoles, restricted to specific tissues / cell types. Tissue-dependent expression levels	Present. Only on some tissues / cells. Tissue-dependent expression timing	Present	–	(Flajnik et al. 1986, 1990; Flajnik and Du Pasquier 1988; Du Pasquier and Flajnik 1990; Gravenor et al. 1995; Rollins-Smith et al. 1996, 1997; Salter-Cid et al. 1998; Izutsu et al. 2000; Mescher et al. 2007; Goyos et al. 2009; May and Beebee 2009; Didinger et al. 2017; Lau et al. 2017, 2020)
Adult	Present. Species-dependent expression levels	Present. Tissue-dependent expression levels. Reduced expression when grown with toxins	–	Higher in spleen and intestine	(Du Pasquier et al. 1974; Flajnik et al. 1984, 1986, 1990; Kaufman et al. 1985b; Flajnik and Du Pasquier 1988; Rollins-Smith and Blair 1990; Harding et al. 1993; Kobari et al. 1995; Gravenor et al. 1995; Rollins-Smith et al. 1996; Salter-Cid et al. 1998; Castell-Rodríguez et al. 1999; Liu et al. 2002; Stewart et al. 2005; Savage et al. 2014; Didinger et al. 2017; Lau et al. 2017, 2020; Neely et al. 2018; Robert et al. 2019; Ma et al. 2020)
*Caudata*					
Larva	From eclosion	Tissue-dependent expression timing	–	–	(Sammut et al. 1997; Völk et al. 1998; Bulut et al. 2008; Babik et al. 2009)
Adult	–	Expressed	–	–	(Babik et al. 2009)

## The Role of MHC in Amphibian Disease

Amphibians are experiencing population crashes and extinctions worldwide, and many of these are related to infectious diseases (Luedtke et al. 2023). Yet, the role of MHC in amphibian disease outcome has only been investigated for specific MHC classes in relation to a few specific diseases (Table 4, Online Resource 1). For instance, most studies on the fungus *Batrachochytrium dendrobatidis* and the bacteria *Aeromonas hydrophila* focus on classical MHC class II, while studies on viruses of the genus *Ranavirus* tend to focus on both classical MHC classes (Table 4, Online Resource 1). Here, only general patterns across all diseases will be discussed, as focused reviews on the immunological aspects of specific amphibian diseases are already available (*Worms*: Tinsley 1995; *Fungi*: Carey et al. 1999; Richmond et al. 2009; Rollins-Smith et al. 2009; Van Rooij et al. 2015; Fu and Waldman 2017; Grogan et al. 2018, 2020; *Viruses*: Carey et al. 1999; Robert 2010; Chen and Robert 2011; Jiang et al. 2021; *Bacteria*: Carey et al. 1999; Hyoe and Robert 2019; Paiola et al. 2023; *Tumours*: Goyos and Robert 2009). Most positively selected sites of the amphibian classical MHC class I and class II genes are located in the PBR (Mable et al. 2015; Didinger et al. 2017; Kosch et al. 2017), which highlights the importance of this region in pathogen recognition. Due to the limited and fragmented information available on the specific MHC codons under selection, it is not possible to make any further generalizations.

**Table 4:** Studies documenting relationships between amphibian health and MHC proteins. No information is available for non-classical MHC class II. For more details see Online Resource 1

		Classical		Non-classical	References
		MHC I	MHC II	MHC I	
Bacteria	Susceptible	–	Related to specific alleles	–	(Barribeau et al. 2008; Zeisset and Beebee 2010)
	Resistant	–	Related to specific alleles	–	(Barribeau et al. 2008; Yu et al. 2014; Hu et al. 2017)
	Other	–	Individuals with resistant alleles grew slower. Related to specific allele presence. More diverse in heterozygotes. No relation	Impairing it increased susceptibility	(Barribeau et al. 2008; Edholm et al. 2018; Hernández-Gómez et al. 2018; Belasen et al. 2021; Hase 2022; Hossainey et al. 2023)
Fungus	Susceptible	Related to specific alleles and supertype	Related to specific alleles/Supertype/Heterozygosity/Conformation. Increased expression in late infection	–	(Bataille et al. 2015; Savage and Zamudio 2016; Edholm et al. 2018; Savage et al. 2018, 2020)
	Resistant	Related to heterozygosity. Higher expression	Related to specific alleles/Homozygosity/Heterozygosity/Conformation	–	(Savage and Zamudio 2011; Kosch et al. 2016, 2019; Savage et al. 2018; Belasen et al. 2019; Ellison et al. 2020; Cortazar-Chinarro et al. 2022; Fu et al. 2023b)
	Tolerant	–	Related to specific alleles, supertype, and conformation	–	(Bataille et al. 2015; Savage and Zamudio 2016)
	Other	Expression patterns vary with temperature and between tissues. Increased expression in infected individuals. Co-infection causes weak upregulation	Heterozygosity related to prevalence. Specific alleles, supertypes, and heterozygosity associated with risk of infection. Less diversity in infected populations. Expression patterns vary with temperature and between tissues	Co-infection causes weak upregulation, lower than single pathogen infection	(Rosenblum et al. 2012, 2009; May et al. 2011; Ellison et al. 2014, 2020; Bataille et al. 2015; Kosch et al. 2016; McDonald et al. 2020; Trujillo et al. 2021; Belasen et al. 2022; LaFond et al. 2022; Becker et al. 2023)
Virus	Susceptible	Related to homozygosity	–	–	(Gantress et al. 2003)
	Resistant	Related to supertype	–	–	(Teacher et al. 2009)
	Tolerant	–	Related to heterozygosity and supertypes	–	(Savage et al. 2019)
	Other	Infection increases expression. Diversity/supertype correlated with pathogen abundance/richness	Infection increases expression. Specific alleles related to infection intensity	Infection increases expression. Impairment increases susceptibility	(Marr et al. 2005; Morales and Robert 2007; Teacher et al. 2009; Edholm et al. 2013, 2019; Zhu et al. 2014; Wang et al. 2017; Chen et al. 2018b; Ke et al. 2018; Savage et al. 2019)
*Trematode*	Resistant	–	Related to diversity	–	(Hernández-Gómez et al. 2019)
Tumour	Other	Some tumours lack expression. Tumour relies on it to avoid immunity. Impairment increases susceptibility	Some tumours lack expression	Present in tumours without MHC class Ia. Fights cancer cells	(Du Pasquier and Robert 1992; Robert et al. 1994; Du Pasquier et al. 1995; Horton et al. 1996; Goyos et al. 2007, 2009; Haynes-Gilmore et al. 2014; Banach and Robert 2019; Banach et al. 2019)
Albinism	Other	Overexpression	–	–	(Chang et al. 2022)

Amphibian resistance to pathogen-induced diseases has been associated with multiple aspects of the classical MHC: (1) specific MHC polypeptide conformations (Bataille et al. 2015; Kosch et al. 2016); (2) specific alleles (Hu et al. 2017; Savage et al. 2018) or supertypes (Savage and Zamudio 2011, 2016; Belasen et al. 2019); and (3) overall heterozygosity (Kosch et al. 2019; Belasen et al. 2019; Hernández-Gómez et al. 2019) (Table 4, Online Resource 1). However, not all individuals with a favourable MHC genotype survive, and not all surviving infected populations harbour increased frequencies of resistance-conferring genotypes. For instance, in *Litoria verreauxii* (Whistling tree frog; Fig. 2i), resistance was associated with homozygosity at a specific conformation of the classical MHC class II PBR in laboratory conditions, while in the wild heterozygosity was the most common state (Bataille et al. 2015). Since amphibians are susceptible to a wide range of pathogens (Kiesecker et al. 2001), MHC heterozygosity might be generally favourable as it broadens the range of pathogens detected (Kohn et al. 2006). This may not hold when populations are facing highly lethal pathogens (Kosch et al. 2016). For instance, in a population of *Lithobates chiricahuensis* (Chiricahua leopard frog; Fig. 2j) under decline due to chytridiomycosis, strong selection for specific resistance-conferring alleles appeared to contribute to overall population survival, but also resulted in reduced MHC allelic diversity (Savage et al. 2018). Furthermore, environmental conditions can influence MHC-based resistance. For instance, in *Plethodon cinereus* (Red-backed salamander; Fig. 2k), certain MHC alleles were found to be beneficial to resistance only in cold conditions (Ellison et al. 2020). This is likely related to temperature-mediated changes in gene expression (Ellison et al. 2020). The MHC genotype can also affect disease outcome indirectly by influencing the skin microbiome community composition (Belasen et al. 2021).

To understand the role of the MHC in mediating pathogenic disease progression, several studies have also quantified classical MHC class I and II glycoprotein expression levels throughout infection with several pathogens (Table 4). Expression levels of classical MHC genes differed between MHC classes, tissues (Ellison et al. 2020; Savage et al. 2020), and pathogens (Ke et al. 2018; McDonald et al. 2020), and they covaried with environmental conditions (Ellison et al. 2020) and the time elapsed since infection (Zhu et al. 2014). For instance, *Notophthalmus viridescens* (Eastern newt) increases gene expression levels of MHC class I genes more when infected with two pathogens than when infected with a single one, but this was not true for other genes of the MHC regions (McDonald et al. 2020). Expression levels of non-classical MHC genes are also known to increase with infection progression (Edholm et al. 2019) and silencing of these genes increases disease susceptibility (Edholm et al. 2018). This can be seen in *X. laevis* where the expression of specific non-classical MHC class I genes increases drastically in infected individuals (Edholm et al. 2019). As research on amphibian MHC expression during disease is still in its infancy, there is not enough evidence to generalize.

For non-pathogenic diseases such as cancer, research tends to focus on the potential role of non-classical MHC class I (Robert et al. 2003, 2009; Table 4; Online Resource 1). The expression of non-classical MHC class I genes on cell membranes allows the immune system to evaluate the functional status of a cell (*i.e.*, normal, infected by pathogens, or cancerous; Banach and Robert 2017), and silencing of these genes results in increased tumorigenesis (Goyos et al. 2007). This is evident in *Xenopus* mutants without non-classical MHC class I genes, where tumours can grow without being detected by the organism (Goyos et al. 2007). *Xenopus* is a particularly interesting and very well-established model system for tumour biology, due to the spontaneous generation of tumours with cells with different MHC class I expression patterns (Du Pasquier and Robert 1992; Robert and Cohen 1998; Luisetto et al. 2019) and sometimes lacking MHC class II expression (Du Pasquier and Robert 1992; Du Pasquier et al. 1995). Studies on these models have revealed that the MHC is critical in tumour immunity and can contribute to the development of new cancer immunotherapies for other vertebrates including humans (Banach and Robert 2017).

## MHC and Amphibian Social Behaviour

In many taxa, MHC genotype plays a role in kin recognition (Manning et al. 1992; Yamazaki et al. 2000). This has been observed in amphibians as well. For example, *X. laevis* tadpoles preferentially school with individuals with similar classical MHC class I and II genotypes (Villinger and Waldman 2008; Table 5) based on PBR sequence similarity (Villinger and Waldman 2008, 2012). This behaviour might be beneficial because individuals with different MHC genotypes are more likely to carry pathogens that the focal individuals are not resistant to (Lewis 1998). When grouped with individuals with dissimilar MHC genotypes, *X. laevis* tadpoles develop faster (without growing larger) and have reduced survival (Barribeau et al. 2012).

**Table 5:** Studies documenting relationships between amphibian behaviour and MHC proteins. No information is available for non-classical MHC class II. For more details see Online Resource 1

	Classical		References
	MHC I	MHC II	
Tadpole	Schooling with similar alleles/siblings. Faster growth and increased mortality with dissimilar individuals	Schooling with similar alleles/siblings. Faster growth and increased mortality with dissimilar individuals	(Villinger and Waldman 2008, 2012; Barribeau et al. 2012)
Adult	–	Heterozygosity did not increase reproductive success. Increased mating success of couples with dissimilar alleles	(Bos et al. 2008, 2009; Luo et al. 2016)

In a range of vertebrate taxa, individuals have been shown to prefer mates with dissimilar classical MHC class I and II genotypes (reviewed in Milinski 2006; Winternitz et al. 2017). For amphibians, such evidence is largely absent (Table 5). In the only study that looked directly at the link between MHC and mate preferences in amphibians, female *Ambystoma tigrinum* (Tiger salamander; Fig. 2l) preferred mating with males with intermediate dissimilarity at the classical MHC class II locus (Bos et al. 2009). This observation is intriguing but requires validation. For instance, preference was inferred from parentage, which was established based on only four microsatellite loci, and not all offspring could be assigned to the experimental males—suggesting that females stored sperm from matings preceding the experiments (Bos et al. 2009). Another approach for detecting MHC-based mating patterns is characterizing the number of different genotypes in natural populations. In *Triturus carnifex* (Italian crested newt; Fig. 2m), genotype diversity tends to be higher than expected based on allele frequencies (Talarico et al. 2019), which could be the result of disassortative mating. More work is required to determine whether MHC genes actually play a role in adult amphibian social interactions and whether the patterns are consistent with observations in other vertebrates. Furthermore, amphibians are an exciting group to study the effects of sexual selection in the evolution of MHC due to the large diversity of mating strategies, even among closely related species. If amphibians show MHC-mediated mate choice, as observed in many other vertebrates (Landry et al. 2001; Bonneaud et al. 2006), this could be a major contributor to MHC evolution in this group and essential for the evolution of disease resistance in natural populations.

## Research Priorities

**Fig. 4:**
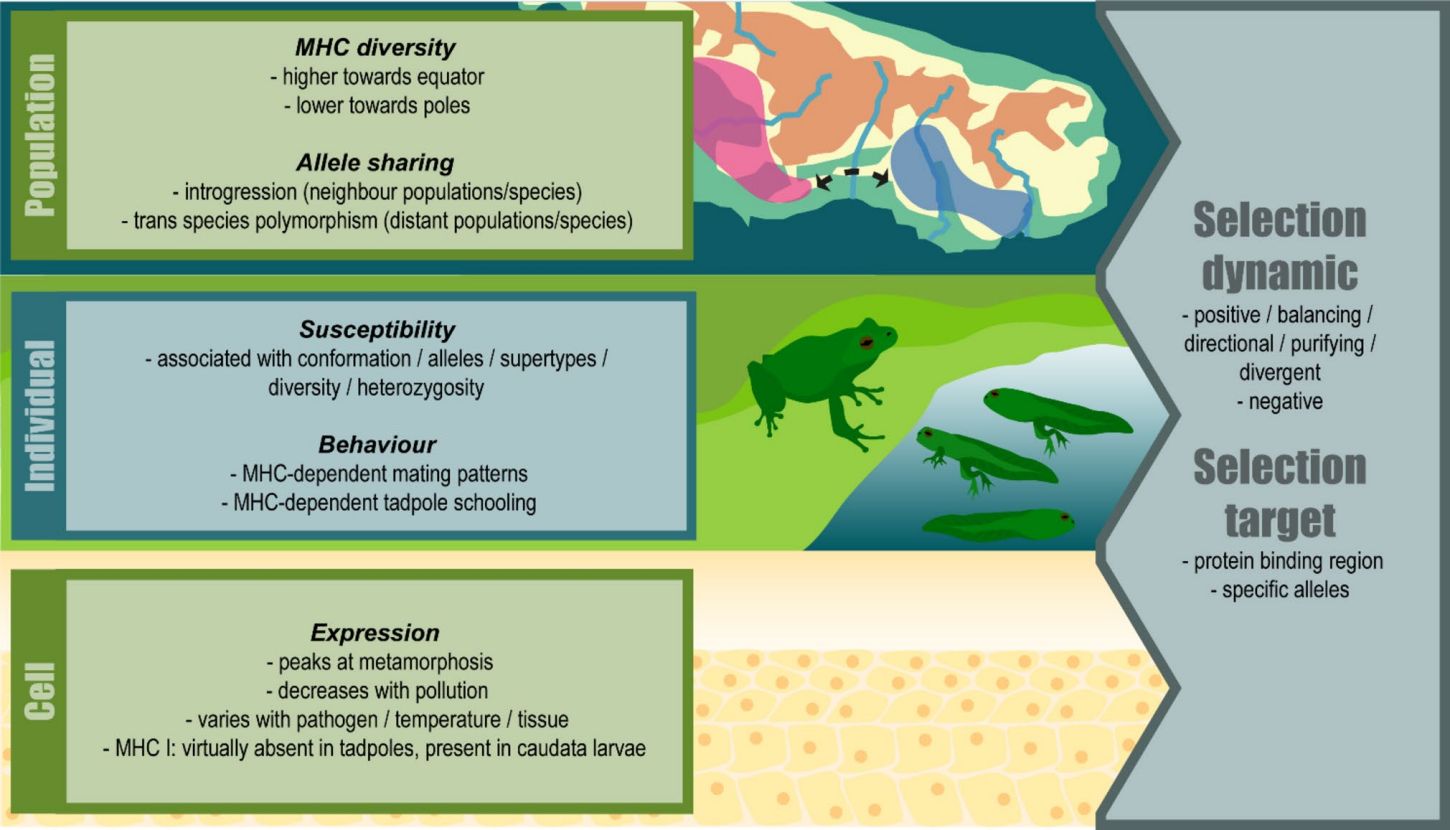
Visual representation of the current state of knowledge regarding the amphibian MHC. On the left, information is divided into three levels of biological organization: Population, Individual, and Cell. On the right, selection dynamics and targets identified in the literature that correlate with the phenomena presented on the left are listed. Figure was created with Adobe Illustrator

In this review, we aimed to summarize the current state of knowledge regarding the amphibian MHC (Fig. 4), and to inspire future research on the evolution and functional significance of this key vertebrate trait. While the first amphibian MHC study was published about 50 years ago (Du Pasquier et al. 1974), the study of the MHC of amphibians is only taking its first steps, especially when compared to other organisms (Kasahara 2000; Mehra et al. 2010). There are still relatively few amphibian MHC studies, covering fewer than 1% of all described amphibian species (Online Resource 1). While the thorough study of a limited set of model organisms is important, it is insufficient for a general understanding of the MHC in amphibians.

The MHC was first described in 1975 (Roux and Volpe 1975), but there are still many fundamental questions that remain unanswered. We propose that amphibians, due to their unique phylogenetic position and diverse life histories, represent a promising model system. Amphibians also have an abundance of invitrome possibilities (Fellah et al. 2002; Douglas et al. 2023) which makes them ideal for enhancing our understanding of MHC evolution and functioning in general. We identify three specific target areas that need to be further explored in the next decade: (1) MHC genomic structure, (2) MHC functioning, and (3) Fitness consequences of MHC variation.

1. *MHC genomic structure.* The structure of the MHC genomic region differs between vertebrate groups, as well as the location of specific genes within the three subregions (Fig. 1; Flajnik and Kasahara 2001; Ohta et al. 2006; Flajnik 2018). The causes and consequences of these different organizations remain unclear. To explore the evolutionary history and functional consequences of alternative configurations, it is necessary to establish MHC configuration in a broad range of taxa. The amphibian MHC in particular, can help fill these gaps as close relatives have different MHC organizations as well as diverse life histories and ecologies. Significant progress has been made in generating data for this specific objective, as several amphibian genome sequencing initiatives are ongoing or under development (Kosch et al. 2024) which will facilitate analyses at the family (or even genus) level in the near future.
2. *MHC functioning*. The presence of specific MHC genes does not guarantee biological function, as exemplified in birds where multiple MHC class I gene copies exist, but expression is dominated by only a few (Drews and Westerdahl 2019). To disentangle gene presence from biological function, we need to explore variation in gene expression across, for instance, tissues, species, and immune activation states. Due to their biphasic lifestyle, amphibians are a suitable target group in which to study patterns of MHC gene expression variation, across developmental stages and in response to exposure to terrestrial vs. aquatic pathogen communities. Finally, amphibians present an exceptional model to explore the intricacies of MHC function because of their additional codon in the classical MHC class II gene (Kiemnec-Tyburczy et al. 2010; Bataille et al. 2015; Mulder et al. 2017) and the extensive allele sharing even between distant species.
3. *MHC genetic variation and fitness*. Genetic variation is the raw material of evolution; however, it is only consequential when it results in differential survival or reproductive success (*i.e.*, fitness). Therefore, it is essential to understand how MHC variation affects individual performance. There are at least three main questions that remain unanswered in this regard: (1) While the functional roles of most genes in the MHC genomic region are known (Murphy et al. 2022), the adaptive significance of the high degree of MHC polymorphism observed across all vertebrates remains uncertain; (2) MHC genes are known to be subject to alternative splicing, however the effect of alternative splice variants of MHC genes, and of the presence or absence of these variants across species is still unexplained; (3) mate preferences for allelic diversity or for specific alleles can play a critical role in shaping the population genotype pool, but how these preferences explain the dynamics of MHC evolution in natural populations is yet not fully understood. Amphibians are a great group in which to address these questions as they exhibit a high degree of MHC diversity between closely related species, possibly related to differences in life history, and they are very sensitive to environmental alterations. Additionally, many amphibians seasonally migrate between terrestrial and aquatic habitats, providing an exceptional opportunity to study pathogen-mediated selection on the MHC, analogous to migratory birds (Minias et al. 2017), but at a much more manageable geographical scale.

To conclude our list of research priorities, we add a methodological recommendation: we call for consistency in the reporting of MHC data. Incomplete data descriptions are common (*e.g.*, number of individuals analysed, number of alleles/sequences found per individual and in total, sex of sampled individuals) and they make it extremely difficult to compare findings across studies and identify general patterns. We recommend that studies should at least clearly report the items mentioned above to facilitate data comparison and integration.

Finally, we posit that recent scientific advances place us in an exciting time to explore the role of MHC in vertebrate evolution. Amphibians provide a great system for these studies due to their phylogenetic position, their biphasic lifestyle and metamorphosis, their multiple evolutionary transitions to water-independent development, their highly diverse mating systems, and the possibilities for research on amphibian cell lines. Hence, studying the amphibian MHC has the potential to expand our general understanding of MHC evolution and functioning, as well as to contribute to the conservation of this charismatic yet extremely threatened taxon.

## Supplementary Information

Below is the link to the electronic supplementary material.

Supplementary file1 (XLSX 90 KB)
